# Microwave ablation of malignant extremity bone tumors

**DOI:** 10.1186/s40064-016-3005-8

**Published:** 2016-08-20

**Authors:** Qing-Yu Fan, Yong Zhou, Minghua Zhang, Baoan Ma, Tongtao Yang, Hua Long, Zhe Yu, Zhao Li

**Affiliations:** Tangdu Hospital, Fourth Military Medical University, 569 Xinsi Road, Baqiao District, Xi’an, Shaanxi China

**Keywords:** Microwave, Ablation, Malignant bone tumor

## Abstract

**Background:**

The current application of limb salvage process has some unsolved problems, such as prosthesis loosening, which severely limits the function of the preserved limbs. Innovative approaches are needed to further improve functional outcome.

**Patients and methods:**

Instead of en-bloc resection of tumor-bearing bone, it is dissected from the surrounding normal tissues, followed by devitalizing the bone segment and the extra-cortical bulk by microwave induced hyperthermia in situ through the antenna array. From May 1999 to March 2012, 544 patients with malignant bone tumors of the extremities were treated by the novel method.

**Results:**

The over 3-year survival rate was 59.1 % for high-grade malignancy, 88.7 % for low-grade malignancy. In the majority of the patients, cosmetic and useful limbs were preserved. Local recurrence rate was 9.8 % for the high grade malignancy (mainly occurred at the early stage of the research). The overall fracture rate was 2.6 %. Deep infection rate was 1.8 %. The complication rate is lower than the literature reports. After heat necrosis, the dead bone maintains both the osteoconduction and osteoinduction properties.

**Conclusions:**

The application of microwave induced hyperthermia for treatment of malignant bone tumors, except the late diagnosed cases who’s tumor-bearing bone was destroyed too severe to do biological reconstruction, is an effective, simple, and inexpensive method. The oncological and functional results are encouraging.

## Background

About thirty-five years ago, malignant bone tumor (mainly osteosarcoma), which often occurred in children, adolescents and young adults, meant amputation and death. More than 90 % of the patients died from pulmonary metastasis. It was once considered such a fatal condition that even the terms of “months to metastasis” were used to describe the oncological outcome (Coventry and Dahlin 1957). With the advent of multi-agent chemotherapy together with improved surgical techniques, long-term survival probabilities for osteosarcoma have improved dramatically during the late twentieth century. In 2002, nearly 80 % of these patients can be cured and their limbs can be saved too (Picci 2007; Rosen 1985).

The main goal of limb salvage surgery for malignant bone tumors is to save the diseased limb through non-mutilating surgery and without jeopardizing the prospects of survival; it has been practiced for more than three decades. This type of operation mainly consists of two parts: en bloc resection of the tumor-bearing bone and reconstruction for the remained defect. Various techniques have been devised for reconstruction, such as metal prosthesis replacement, osteoarticular allograft transplantation, or re-implantation of the autoclaved resected tumor bone (Mankin et al. 1976; Marcove et al. 1977; Veth et al. 2003; Wodajo et al. 2003). But, it is still the subject of debate. Metal prosthesis replacement has been widely used, and proved to be an effective practice. However, long-term complications of the implant and related-bone and late infection are unsolved problems (Avedian et al. 2010; Muscolo et al. 2004). The aseptic loosening at a specified future date is a main concern. Malignant bone tumor tends to occur in adolescents and young adults. For these young people, prosthesis is not satisfying completely since loosening possibly needs to be revised again and again in their life time.

The inherent problems of prosthesis replacement are related to the materials used and the anchorage of metallic parts to bone and to soft tissues. For young patients, the implantation of artificial material will have to stand wear and tear for decades.

The microwave-induced hyperthermia is used to deal with malignant bone tumors for 20 years in our Department. The results from both oncological and functional point of views were encouraging.

## Methods

**Fig. 1:**
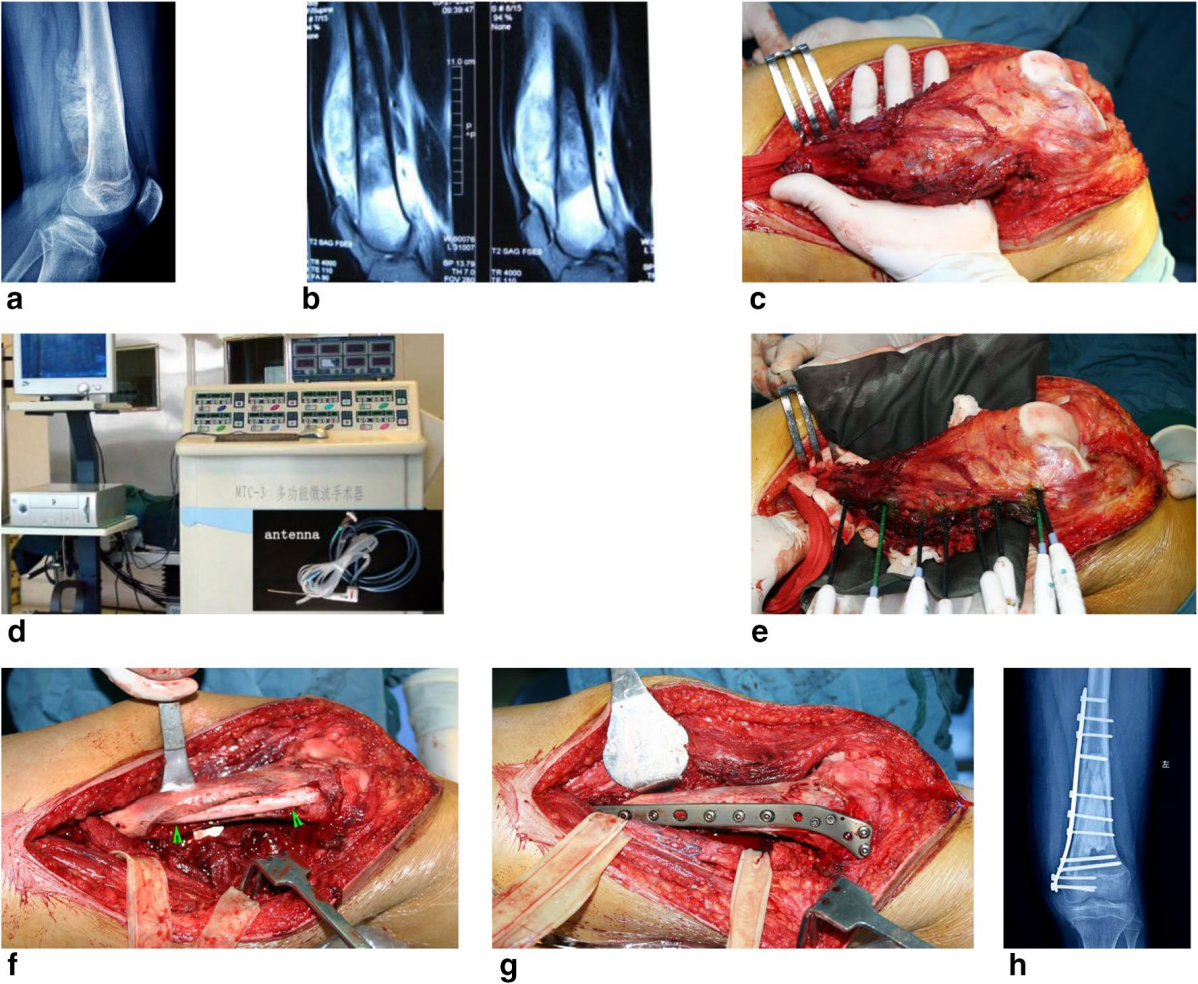
Typical procedure for osteosarcoma at distal femur. **a**, **b** Image data shows an osteosarcoma of distal femur. **c** Dissect the tumor-bearing bone from surrounding normal tissues with safe margin. **d** Put a heat-isolation pad between the tumor bone and surrounding normal tissues and began to deliver electromagnetic energy into tumor bone. **e** The microwave generator and antenna. **f** Remove or curettage the dead soften tumor mass and give fibular bone autograft. The mixture materials of bone chips and bone cement was used filling the cavity. **g** Restore the normal shape of the femur and give a prophylactic fixation. **h** X-ray film after surgery

**Fig. 2:**
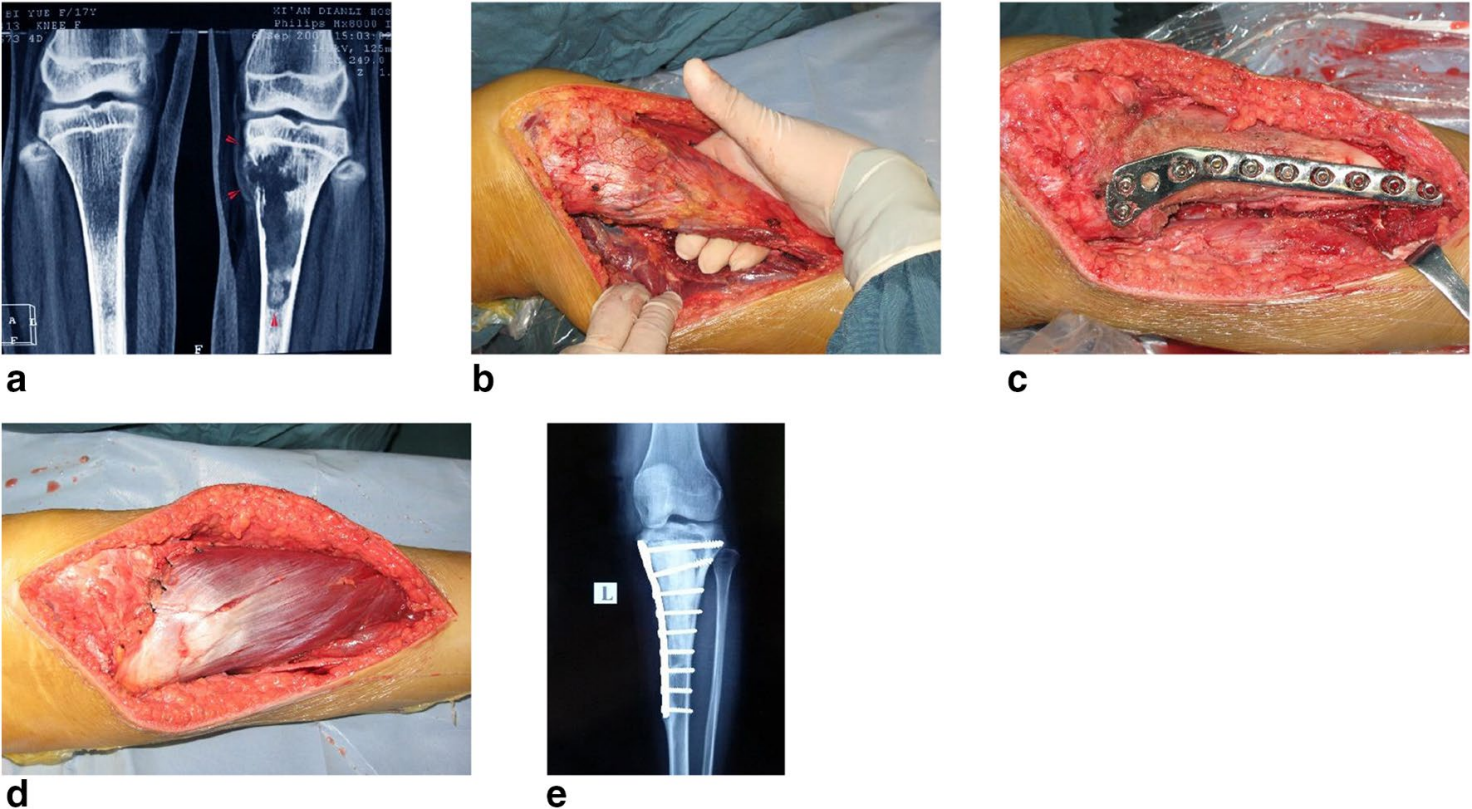
Typical procedure for osteosarcoma at proximal tibia. **a** X-ray film before surgery. **b** Isolation of tumor bone. **c** Give a prophylactic fixation after MWA. **d** Transfer the medial head of gastrocnemius muscle. **e** X-ray film after surgery

The main concept here is to achieve a tumor en bloc ablation with safe margin using antenna-guided hyperthermia therapy. Microwave generator is made in China with 2450 Hz. Currently, co-axial MW antennas (require circulating water to cool the tip of the antenna to avoid metal melting damage due to high temperature) were inserted into the carefully dissected and isolated tumor tissue block. The goal of thermal ablations to create an ablation zone that extends 1 cm beyond the tumor boundary at all points after being heated to 70–100 °C for 20–30 min. During surgery, multiple thermocouples were placed in various critical locations to monitor the temperature within and around the bulk. These dead tissues were removed and/or curetted leaving behind the defective bone for reconstruction using any of the currently accepted methods, such as autograft, allograft, or cementation. If the traditionally defined wide margin could be identified using en bloc resection technique, MW ablation (MWA) can achieve a similar goal while retaining the curetted cortical bone intact, thus making reconstruction easier and more durable. Two examples were given for the tumors at distal femur and proximal tibia, the most common sites of osteosarcoma (Figs. 1, 2; Table 1).

**Table 1:** Clinical data: diagnosis

Classification	Name	Total number
High grade malignancy	Osteosarcoma	435
	Ewing’s sarcoma	29
	Malignant fibrous histiocytoma	5
Low grade malignancy	Chondrosarcoma	38
	Adamantinoblastoma	16
Metastasis		21

**Fig. 3:**
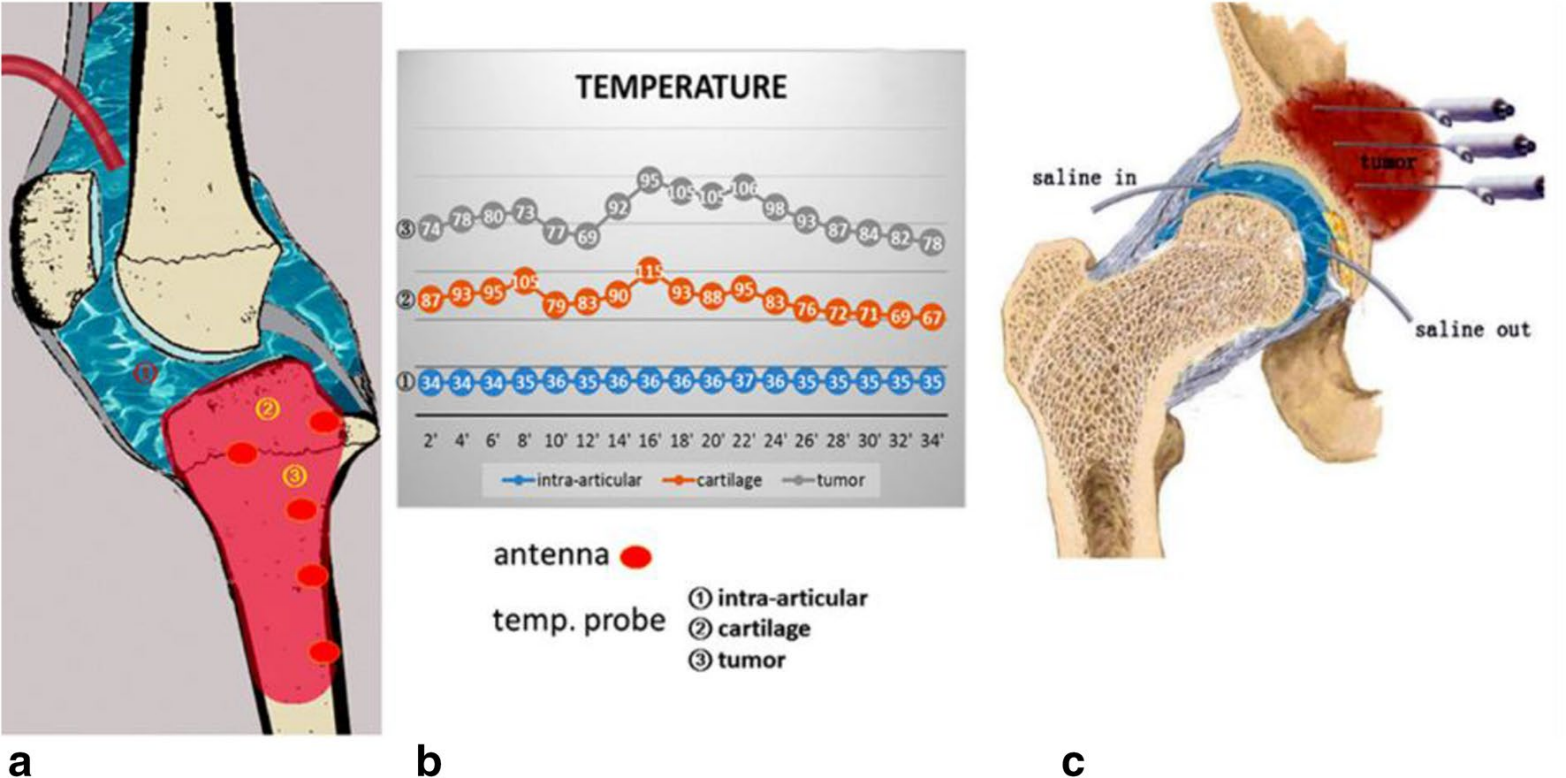
Schematics show how to cooling the cartilage while MWA. **a** Knee joint infusion, **b** temperature curve recorded during surgery, **c** same methord could be used for hip joint

When the lesion is close to the joint but not within the joint , this technique could be used with special cooling system to protect the cartilage, thus saving the joint. Incert a tube into knee joint to infuse cooling saline to protect the cartilage from overheating (Fig. 3).

### Patients

Between July 1999 to March 2012, 544 patients with malignant bone tumors of the extremities were treated using the microwave hyperthermia technique at the authors’ institution.

The 469 cases with high-grade malignant sarcoma (mainly osteosarcoma followed by MFH and Ewing’s sarcoma), 54 cases with low-grade malignancy (chondrosarcoma 38 cases, adamantinoblastoma 16 cases), and 21 cases with isolated metastatic lesion. Distributions according to age, sex, and anatomical location were comparable to that reported in the literature. For high-grade sarcomas, three courses of preoperative chemotherapy were given.

## Results

The greater than 3-year survival rate was 59.1 % for high-grade malignancy. The beyond 3-year survival rate was 88.7 % for low-grade malignancy. For the metastatic lesions, the aim of the surgery was to relieve pain and improve quality of life.

In all surviving patients, their normal posture and activities of daily life were preserved. Nine patients got degeneration of knee joint, and two patients got hip joints degeneration. The mean Musculoskeletal Tumor Society score at last available follow-up was 26. Average functional score was 92 %.

### Complications

Local recurrence rate was 9.8 % (46 patients) for the high grade malignancy (mainly occurred at the early stage of the research). 9 patients’ limbs were preserved after reoperation, The 35 patients received amputation. The overall fracture rate was 2.6 %. They were treated by revision surgery as do for the general fracture patients. Deep infection rate was 1.8 %. The infection got controlled by revision surgery.

## Discussion

**Fig. 4:**
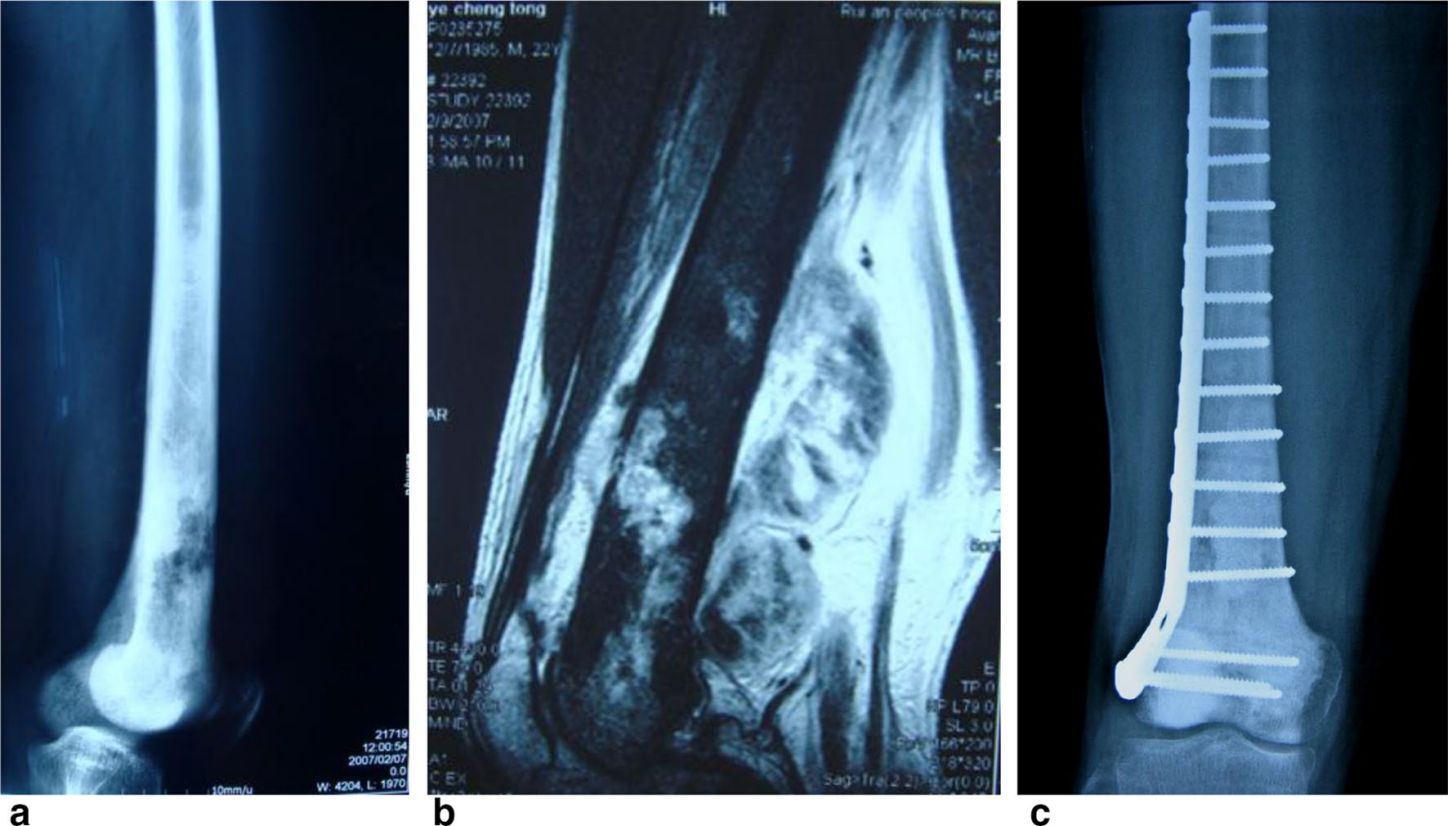
A patient with osteosarcoma involving nearly 3/4 length of the femur. The function is excellent after surgery for more than 10 years. **a** X-ray film showing the lesion involved the majorlity of the femur, **b** MRI showing the huge tumor bulk, **c** X-ray film post-operation, the function is perfect at 10 years after surgery

It is generally accepted that although the limb salvage surgery is originally successful, it could be a failure with time. The overall event-free prosthesis survival was 63 % at 5 years and 36 % at 10 years (Horowitz et al. 1993). About 70 % of patients at 10 years required further surgical procedures and reported a 25 % risk of amputation (Grimer et al. 1999). In the short- and intermediate-term these surgeries have been associated with relatively good function and low revision rates. However, long-term studies show high rates of soft tissue, implant, and bone-related complications (Avedian et al. 2010; Muscolo et al. 2004). The function result after micronave ablation is durable as the case showing in Fig. 4.

### About microwave ablation (MWA)

Temperature is one of the most important environmental factors for all of living cells. The upper temperature limit for plants and animals is <50 °C (120 °F). Between 60–100 °C, there is near instantaneous induction of protein coagulation which irreversibly damages key cytosolic and mitochondrial enzymes, as well as nucleic acid–histone protein complexes (Goldberg et al. 2000).

Thus, a key aim for ablative therapies is achieving and maintaining a 60–100 °C temperature range throughout the entire target volume. Microwave energy, typically at 915 MHz or 2.45 GHz, radiated by the antenna causes rapid rotation of water, proteins and other polar molecules in tissue, a process known as dielectric hysteresis. Microwaves enable faster heating of larger targets.

Advances in diagnostic imaging techniques permit an accurate preoperative determination of the tumor extent (Muscolo et al. 2004). The devitalized extension of bone depends upon the image data, especially the MIR data.

To reduce the risk of tumor prosthesis breakage, the amount of bone resection should be limited to 30 % or less in the affected bone. The tumor involved 2/3 femur in the following case showed in Fig. 4. It can be treated by MWA successfully.

#### *About the fate of dead bone*

Previous researches have showed that the microwave ablation decreased bone stiffness but no change in the breaking load, and also no serious effect to revascularization and new bone formation (Ji et al. 2012; Kawaguchi et al. 2004; Quan et al. 2005). That is mean that after heat necrosis,the dead bone maintains both the osteoconduction and osteoconduction properties. The devitalized osseous structure, like an autogenous bone graft, can be used as biological scaffold for reconstruction. Once healing occurred, it is durable, so there is no worry about wear and tear as to the prosthesis. If it endures the first year, this suggests that it will thereafter remain functional for the duration of patient’s life. But, radiological remodeling of the devitalized bon was not complete. This means that framework with partial dead bone remained thus needing the long-term support of the metalwork.

The functional outcome is expected to be superior after MW ablation, since no joint replacement—neither prosthetic nor allograft—could function better than an intact native joint (Ceruso et al. 2008; Fuchs et al. 2008; Thompson et al. 2000).

The devitalized bone allows re-attachment of muscles and tendons. The natural knee joint was retained perfectly.

In a few cases, the tumor-bearing bone was destroyed so severe that the remainder of the bone stock is not enough to restore its mechanical strength even after reconstruction, other methods (such as prosthesis replacement, allograft, or extracorporeal devitalization and replantation) were used for limb salvage.

The advantage of MWA can be concluded:

1. It improves the function outcome
2. The function is durable
3. It greatly simplifies surgery process for pelvic tumors
4. It is lowers costs

Even though our 11-year experience has not been uniformly satisfactory, pretty sufficient success has been achieved. Advances in surgical skill and antenna design have made microwave ablation procedures a realistic option for limb salvage without compromising survival or local recurrence. Hyperthermia should deserve more attention than it has received until now.

## References

[CR1] Avedian RS, Haydon RC, Peabody TD (2010). Multiplanar osteotomy with limited wide margins: a tissue preserving surgical technique for high-grade bone sarcomas. Clin Orthop Relat Res.

[CR2] Ceruso M, Taddei F, Bigazzi P, Manfrini M (2008). Vascularised fibula graft inlaid in a massive bone allograft: considerations on the bio-mechanical behaviour of the combined graft in segmental bone reconstructions after sarcoma resection. Injury.

[CR3] Coventry MB, Dahlin DC (1957). Osteogenic sarcoma; a critical analysis of 430 cases. J Bone Joint Surg Am.

[CR4] Fuchs B, Ossendorf C, Leerapun T, Sim FH (2008). Intercalary segmental reconstruction after bone tumor resection. Eur J Surg Oncol.

[CR5] Goldberg SN, Gazelle GS, Compton CC, Mueller PR, Tanabe KK (2000). Treatment of intrahepatic malignancy with radiofrequency ablation: radiologic-pathologic correlation. Cancer.

[CR6] Grimer RJ, Carter SR, Tillman RM, Sneath RS, Walker PS, Unwin PS, Shewell PC (1999). Endoprosthetic replacement of the proximal tibia. J Bone Joint Surg Br.

[CR7] Horowitz S, Glasser D, Lane J, Healey J (1993). Prosthetic and extremity survivorship after limb salvage for sarcoma. How long do the reconstructions last?. Clin Orthop Relat Res.

[CR8] Ji Z, Ma Y, Li W, Li X, Zhao G, Yun Z, Qian J, Fan Q (2012). The healing process of intracorporeally and in situ devitalized distal femur by microwave in a dog model and its mechanical properties in vitro. PLoS ONE.

[CR9] Kawaguchi N, Ahmed AR, Matsumoto S, Manabe J, Matsushita Y (2004). The concept of curative margin in surgery for bone and soft tissue sarcoma. Clin Orthop Relat Res.

[CR10] Mankin HJ, Fogelson FS, Thrasher AZ, Jaffer F (1976). Massive resection and allograft transplantation in the treatment of malignant bone tumors. N Engl J Med.

[CR11] Marcove RC, Lewis MM, Rosen G, Huvos AG (1977). Total femur and total knee replacement. A preliminary report. Clin Orthop Relat Res.

[CR12] Muscolo DL, Ayerza MA, Aponte-Tinao LA, Ranalletta M (2004). Partial epiphyseal preservation and intercalary allograft reconstruction in high-grade metaphyseal osteosarcoma of the knee. J Bone Joint Surg Am.

[CR13] Picci P (2007). Osteosarcoma (osteogenic sarcoma). Orphanet J Rare Dis.

[CR14] Quan GM, Slavin JL, Schlicht SM, Smith PJ, Powell GJ, Choong PF (2005). Osteosarcoma near joints: assessment and implications. J Surg Oncol.

[CR15] Rosen G (1985). Preoperative (neoadjuvant) chemotherapy for osteogenic sarcoma: a ten year experience. Orthopedics.

[CR16] Thompson RC, Garg A, Clohisy DR, Cheng EY (2000). Fractures in large segment allografts. Clin Orthop Relat Res.

[CR17] Veth R, van Hoesel R, Pruszczynski M, Hoogenhout J, Schreuder B, Wobbes T (2003). Limb salvage in musculoskeletal oncology. Lancet Oncol.

[CR18] Wodajo FM, Bickels J, Wittig J, Malawer M (2003). Complex reconstruction in the management of extremity sarcomas. Curr Opin Oncol.

